# Efficacy of Bamlanivimab in Reducing Hospitalization and Mortality Rates in COVID-19 Patients in a Rural Community

**DOI:** 10.7759/cureus.16477

**Published:** 2021-07-19

**Authors:** Leena Iqbal, Thomas J Terlau, Alexander Hernandez, Kenneth Woods

**Affiliations:** 1 Emergency Medicine, Trinity West Medical Center, Steubenville, USA; 2 Infectious Disease, Trinity West Medical Center, Steubenville, USA

**Keywords:** bamlanivimab, covid-19, hospitalization in covid-19, comorbidities and covid-19, sars-cov-2

## Abstract

Objective

In this study, we aimed to evaluate the association between the monoclonal antibody infusion, bamlanivimab, and a reduction in 30-day hospitalization and mortality rates in patients with coronavirus disease 2019 (COVID-19) in a rural community setting with various comorbidities.

Methodology

A retrospective data analysis was conducted over a 60-day period for patients who visited the Emergency Department of a community hospital. A group of COVID-19-positive patients who received bamlanivimab was compared with another group of COVID-19 patients with similar characteristics, demographics, and disease severity who did not receive the infusion. Data for 30-day hospitalization rates were analyzed using odds ratio for various individual comorbidities. Fisher’s exact test was used to analyze chronic kidney disease (CKD) and mortality. Logistic regression analysis and subsequent odds ratio estimates were used to adjust for demographics and comorbidities and evaluate for the association of bamlanivimab infusion with 30-day hospitalization rates.

Results

A total of 144 patients were included in the bamlanivimab group and 140 patients in the non-bamlanivimab group of COVID-19 patients. When analyzed by comorbidity using odds ratio analysis, 10.3% of diabetic patients, 6.1% of obese patients, 8% of hypertensive patients, and none of the patients with CKD required hospitalization at 30 days from the initial visit in the bamlanivimab group in contrast to 35.2% of diabetic patients, 38.1% of obese patients, 33.9% of hypertensive patients, and 63.6% of patients with CKD in the non-bamlanivimab group. Logistic regression analysis with odds ratio estimates showed that when adjusted for demographics and various comorbidities, bamlanivimab infusion was associated with decreased hospitalization (p < 0.001; confidence interval [CI] = 0.017-0.135). However, using logistic regression and taking all variables into account, among the evaluated comorbidities, only hypertension was found to be individually associated with decreased hospitalization (p = 0.0174; CI = 0.140-0.827) along with younger age (p = 0.0023; CI = 1.017-1.080) and female gender (p = 0.0077; CI = 0.212-0.789). We could not establish mortality benefit in the subgroups.

Conclusions

Based on the results of this study, there is an association between bamlanivimab use and reduced hospitalization rates in COVID-19 patients.

## Introduction

In December 2019, a new coronavirus was identified after cases of viral pneumonia emerged in the Hubei province of China. The virus was named severe acute respiratory syndrome coronavirus 2 (SARS-CoV-2) and the disease it causes was named novel coronavirus disease 2019 (COVID-19) [1]. COVID-19 affects different populations in varying severity and is thought to be related to individual response as well as demographics and comorbidities [2]. Several treatments have emerged with only very few granted emergency use authorization (EUA) by the Food and Drug Administration (FDA) in the United States. In October 2020, the investigational neutralizing IgG1 monoclonal antibody bamlanivimab (LY-CoV555; Lilly) was granted EUA for use in mild-to-moderate COVID-19 patients with select demographics and risk factors in the outpatient or emergency department setting as a single one-time intravenous infusion [3]. The BLAZE-1 trial supported the use of bamlanivimab and showed a significant reduction in viral load in patients after 11 days compared to placebo [4]. The EUA comes with the rationale that bamlanivimab may reduce COVID-19-related hospital admission or emergency room visits in patients at high risk for disease progression in the 28 days after treatment compared with placebo. The EUA does not mention mortality benefit but may have biological plausibility if the disease progression is interrupted by bamlanivimab infusion. Our study aimed to evaluate any association in mortality benefit or reduction in the risk of hospitalization in patients who received bamlanivimab compared to a similar population of patients with COVID-19 who did not receive bamlanivimab. As a secondary analysis, our study evaluated for any difference in the outcome of patients with diabetes, obesity, hypertension, or chronic kidney disease (CKD) who received the antibody infusion versus patients with similar demographics who did not receive the infusion. By analyzing the data, we hope to give a clearer perspective to the medical community and patients regarding this therapy.

## Materials and methods

Study design

This is a retrospective data analysis conducted from November 12, 2020 to January 10, 2021 over a 60-day period. Patients who visited the Emergency Department (ED) of our community hospital or received an infusion in the infusion center of the community hospital were included in the study. After the protocol was developed, the case was submitted to the institutional review board (IRB) for review. The IRB review determined this project to be exempt from Human Subjects Research according to federal regulations. Two datasets were obtained for comparison. The first dataset was obtained from the pharmacy department at the community hospital where a record was kept of patients who had received the bamlanivimab infusion and had been followed up at two-week and four-week intervals after the infusion according to the institutional protocol. Another dataset was obtained from the ED which included patients with similar demographics as the bamlanivimab group who had been evaluated in the ED during the same time period but were not treated with bamlanivimab. Patients were only included if they were SARS-CoV-2-positive and discharged home. Data included whether they returned for hospitalization or died within 14 or 30-day post-visit. Data were obtained by the pharmacists through follow-up phone calls to the patients as well as chart review looking for re-admission. All data were de-identified before being received by the research team. Data collected were evaluated for any association between treatment with bamlanivimab and subsequent hospital admission or death within 30 days. Secondary outcomes included evaluation for an association of infusion with 30-day hospitalization in patients with specific comorbidities that included diabetes mellitus, obesity, hypertension, or CKD who did or did not receive the antibody infusion.

Inclusion and exclusion criteria

The inclusion and exclusion criteria for the study were similar to the patient population that was approved for the emergency use of bamlanivimab [3]. One exception was pregnant patients who were not included in the study. Both groups had similar demographic criteria.

Inclusion Criteria

Adults 18 years or older with a COVID-19-positive test and one of the additional criteria. Additional criteria included body mass index (BMI) ≥35, CKD, diabetes, immunosuppressive disease and/or receiving immunosuppressive treatment, elevated inflammatory markers (ferritin >500 µg/L, C-reactive protein >50 mg/L, lactate dehydrogenase >245 U/L, and/or absolute lymphocyte count <800/µL), age ≥65 years, age ≥55 years, and presence of cardiovascular disease or hypertension or chronic obstructive pulmonary disease/other chronic respiratory diseases.

Exclusion Criteria

Patients 17 years or younger, pregnant women, patients without laboratory-confirmed COVID-19-positive polymerase chain reaction test, and patients who did not fall into the group mentioned in the inclusion criteria.

Data collection

Data were obtained from the pharmacy and ED after IRB approval was obtained. A data extraction tool in excel was used. Data included age, symptom onset date, date of positive COVID-19 test, qualifying criteria (comorbidities listed in inclusion criteria), BMI, white blood cell count, absolute lymphocyte count, ferritin, C-reactive protein, lactate dehydrogenase, bamlanivimab infusion date, 30-day repeat encounter, hospital admission within 14 days, hospital admission within 30 days, and 30-day mortality. Researchers only received de-identified data with all patient identifiers removed. COVID-19-positive patients with similar demographics who did or did not receive bamlanivimab infusion between November 12, 2020 to January 10, 2021 were included in the study. The sample size consisted of 144 COVID-19-positive patients in the bamlanivimab group and 140 COVID-19 positive patients in the non-bamlanivimab group.

Data analysis

The data obtained were used to compare hospitalization and mortality in the COVID-19-positive bamlanivimab versus non-bamlanivimab group as well as to compare the subgroups of patients with specific comorbidities. All statistical analysis was performed using SAS version 9.4 (SAS Institute Inc., Cary, NC) data analytic software. The odds ratio was used for individual comorbidities including obesity, diabetes mellitus, and hypertension to determine the association between the infusion and 30-day hospitalization. Fisher’s exact test was used for CKD patients and to analyze 30-day mortality. Logistic regression analysis with odds ratio estimates was used to adjust for demographics such as age and gender and comorbidities such as diabetes, obesity, hypertension, and CKD, as well as to assess for the association of bamlanivimab infusion with 30-day hospitalization.

## Results

In this study, we analyzed patients into two groups. A treatment group that received bamlanivimab and a control group that received care without bamlanivimab. Patients in the control arm were eliminated if they required admission on initial presentation or if they required supplemental oxygen. As these patients would not have qualified for the antibody infusion based on the FDA criteria, they were excluded to maintain a similar study population between both groups. After exclusion, 140 patients were included in the non-bamlanivimab group and 144 in the bamlanivimab group. Among the patient population, age ranged from 25 to 95 years. The average age was 66.4 and the median age was 68 (SD = 13.5). Overall, 53% of patients treated with bamlanivimab were female compared to 52% in the group that did not receive the infusion. Both groups had similar demographics and mild disease severity at the time of evaluation in the ED.

The 30-day hospitalization rate

The 30-day hospitalization rates were primarily analyzed using odds ratios (with a 95% confidence interval [CI]) for individual comorbidities. However, the hospitalization rate for CKD patients was analyzed using Fisher’s exact test because there were zero hospitalizations within the bamlanivimab group of patients with CKD. All data were subsequently analyzed using logistic regression analysis with odds ratio estimates adjusted for demographics and comorbidities.

Patients in the group that received the bamlanivimab infusion had a significantly lower 30-day hospitalization rate compared to patients who did not receive the infusion. When stratified based on comorbidities, all groups were found to have a significantly lower rate of 30-day hospitalization. Across all patients, 9/144 (6.3%) who received the bamlanivimab infusion compared to 55/140 (39.3%; 95% CI = 0.048-0.219) who did not receive the infusion were hospitalized within 30 days. When analyzed by comorbidity, 4/39 (10.3%) diabetic patients, 2/33 (6.1%) obese patients, 2/25 (8.0%) hypertensive patients, and 0/20 CKD patients who received the bamlanivimab infusion required hospitalization within 30 days. This is significantly less when compared to those with the same comorbidities who did not receive bamlanivimab with 19/54 (35.2%; 95% CI = 0.065-0.682) diabetic patients, 16/42 (38.1%; 95% CI = 0.022-0.499) obese patients, 38/112 (33.9%; 95% CI = 0.038-0.757) hypertensive patients, and 7/11 (63.6%; p-value = 0.0001) CKD patients requiring hospitalization within 30 days of the initial presentation to the ED.

Table 1 compares 30-day hospitalization rates of COVID-19 patients in the bamlanivimab group versus the non-bamlanivimab group using odds ratio. It also compares different subgroups including COVID-19 patients with diabetes, hypertension, and obesity. Table 2 provides a similar comparison in patients with CKD using Fisher’s exact test.

**Table 1:** 30-day hospitalization rates in COVID-19 patients in the bamlanivimab versus the non-bamlanivimab group and different subgroups based on comorbidities. CI: confidence interval; COVID-19: coronavirus disease 2019

		30-day hospitalization rate			
Group	Total (N)	Bamlanivimab infusion	No infusion	Odds ratio (95% CI)	Sig.
All subjects	284	9/144 (6.3%)	55/140 (39.3%)	0.103 (0.048-0.219)	*
Diabetic patients	93	4/39 (10.3%)	19/54 (35.2%)	0.211 (0.065-0.682)	*
Obese patients	75	2/33 (6.1%)	16/42 (38.1%)	0.105 (0.022-0.499)	*
Hypertension patients	137	2/25 (8.0%)	38/112 (33.9%)	0.169 (0.038-0.757)	*

**Table 2:** 30-day hospitalization rate among COVID-19 patients with CKD in the bamlanivimab versus the non-bamlanivimab group. COVID-19: coronavirus disease 2019; CKD: chronic kidney disease

		30-day hospitalization rate		Fisher’s exact test	
Group	Total (N)	Bamlanivimab infusion	No infusion	P-value	Sig.
CKD patients	31	0/20 (0.0%)	7/11 (63.6%)	0.0001	*

**Figure 1:**
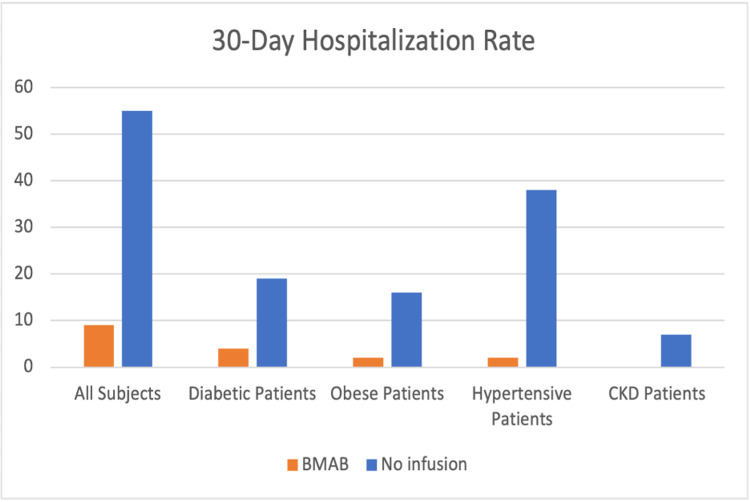
30-day hospitalization rates in the bamlanivimab versus the non-bamlanivimab group of COVID-19 patients. BMAB: bamlanivimab; COVID-19: coronavirus disease 2019; CKD: chronic kidney disease

In all comorbidity subgroups, there were reduced 30-day hospitalization rates for the bamlanivimab group. Figure 1 illustrates the comparison of 30-day hospitalization in different subgroups using a bar graph.

Data were also analyzed using logistic regression analysis with odds ratio estimates for all variables including demographics such as age and gender and comorbidities such as obesity, diabetes, CKD, and hypertension (Table 3). Even when adjusting for age, gender, obesity, and other comorbidities, bamlanivimab infusion was associated with a decreased rate of hospitalization at 30 days from the initial visit (logistic OR = 0.048; 95% CI = 0.017-0.135). Younger age and female gender appeared to have played a significant role in decreased hospitalization with a p-value of 0.0023 and 0.0077 and CI of 1.017-1.080 and 0.212-0.789, respectively. When adjusted for all variables, among the comorbidities, hypertensive patients were found to benefit the most from bamlanivimab infusion with a decrease in 30-day hospitalization (p-value = 0.0174; CI = 0.140-0.827). There was no evidence to suggest an individual benefit of patients with diabetes, obesity, and CKD from bamlanivimab infusion when all other variables were adjusted.

**Table 3:** Logistic regression analysis with odds ratio estimates predicting the likelihood of 30-day hospitalization for all patients. BMAB: bamlanivimab; CKD: chronic kidney disease; DF: degree of freedom

Parameter		DF	Estimate	Standard error	Wald Chi-square	P-value	Odds ratio estimate	95% Wald confidence limits		Sig.
Intercept		1	-4.6693	1.1027	17.9313	< .0001>				
Age		1	0.0470	0.0154	9.2723	0.0023	1.048	1.017	1.080	*
Gender	F	1	-0.4472	0.1678	7.1025	0.0077	0.409	0.212	0.789	*
Obesity	Y	1	0.2678	0.2111	1.6103	0.2044	1.709	0.747	3.908	
Diabetes	Y	1	0.0052	0.1794	0.0008	0.9769	1.010	0.500	2.041	
CKD	Y	1	0.0623	0.2768	0.0507	0.8218	1.133	0.383	3.352	
Hypertension	Y	1	-0.5393	0.2268	5.6527	0.0174	0.340	0.140	0.827	*
BMAB infusion	Y	1	-1.5230	0.2658	32.8353	< .0001>	0.048	0.017	0.135	*

The 30-day mortality rate

Because there were zero deaths within the bamlanivimab group, each measure was analyzed using Fisher’s exact test. Table 4 shows the comparison of 30-day mortality among COVID-19 patients in the bamlanivimab versus the non-bamlanivimab group as well as the subgroups based on comorbidities including diabetes, obesity, hypertension, and CKD.

**Table 4:** 30-day mortality in COVID-19 patients in the bamlanivimab versus non-bamlanivimab group and different subgroups based on comorbidities. BMAB: bamlanivimab; CKD: chronic kidney disease; COVID-19: coronavirus disease 2019

		30-day mortality rate		Fisher’s exact test	
Group	Total (N)	BMAB infusion	No infusion	P-value	Sig.
All subjects	284	0/144 (0.0%)	2/140 (1.4%)	0.2421	
Diabetic patients	93	0/39 (0.0%)	0/54 (0.0%)	***	
Obese patients	75	0/33 (0.0%)	0/42 (0.0%)	***	
Hypertension patients	137	0/25 (0.0%)	1/112 (0.9%)	0.8175	
CKD Patients	31	0/20 (0.0%)	0/11 (0.0%)	***	

None of the groups showed an association of bamlanivimab infusion with a reduced mortality rate. However, this is likely due to the relatively small sample size of this study.

## Discussion

The novel nature of SARS-CoV-2 has made treatment very challenging. The monoclonal antibody infusion, bamlanivimab, was approved for emergency use based on studies that showed success only at reducing viral load. However, there were no studies demonstrating how that translated into clinical outcomes. Given its theoretical benefit based on early data and an acceptable safety profile, bamlanivimab was an early exciting potential option to help reduce the burden of morbidity during this pandemic. With this study, we sought to expand on the limited data regarding the clinical efficacy of bamlanivimab and to explore if it could reduce inpatient care burden and admissions for the treatment of patients with COVID-19 in a rural community hospital setting.

Data analysis aimed to determine mortality benefit and decrease in hospitalization. We could not demonstrate an association between treatment with bamlanivimab and decreased mortality, potentially due to the small sample size. This can be further explored in large multicenter trials with large sample size. This study was able to demonstrate an association between patients who received an infusion of bamlanivimab and a decrease in 30-day hospitalization rates compared to patients who did not receive the infusion.

Multiple published studies have shown that various comorbidities contribute to the severity of COVID-19 and have indicated poor prognosis [5-7]. A recently published large COVID-19 study in England suggested a significantly higher hospitalization and mortality rate. One-third of all in-hospital deaths in patients with COVID-19 were among patients with diabetes [8]. Our study saw a hospitalization rate of 4/39 (10.3%) in diabetic patients in the bamlanivimab group versus 19/54 (35.2%) with a 95% CI of 0.065-0.682 among patients in the non-bamlanivimab group. This is a very promising result for one of our most vulnerable populations for COVID-19.

A retrospective study of COVID-19 in 24 healthcare organizations across the United States found comorbidities such as cardiovascular disease and CKD to be associated with higher mortality and morbidity [2]. Our study showed that hypertension increases the risk of hospitalization in COVID-19 patients. In the bamlanivimab group, 2/25 (8.0%) hypertensive patients required hospitalization. This is in contrast with the 38/112 (33.9%, 95% CI = 0.038-0.757) hypertensive patients who did not receive bamlanivimab therapy. When controlling for comorbidities and other demographic variables, hypertensive patients were found to benefit the most from bamlanivimab infusion with a decrease in 30-day hospitalization (p-value = 0.0174; CI = 0.140-0.827). There was also a significant difference in patients with CKD where no hospitalizations were found in the group of COVID-19 patients who received bamlanivimab therapy compared to 7/11 (63.6%) patients who were hospitalized in the non-bamlanivimab group. Obesity is accepted by the Centers for Disease Control and Prevention as a well-established risk factor for complications with COVID-19 [9]. Our study showed a benefit of bamlanivimab therapy in obese patients (BMI ≥35) with a hospitalization rate of 2/33 (6.1%) obese patients with COVID-19 versus 16/42 (38.1%; 95% CI = 0.022-0.499) obese patients in the non-bamlanivimab group. However, most significantly, we found that when adjusting for age, gender, obesity status, and other comorbidities, bamlanivimab infusion was associated with a decreased rate of hospitalization at 30 days from the initial visit for all patients who qualified for the study (logistic OR = 0.048; 95% CI = 0.017-0.135). Therefore, although we cannot say that any one of the comorbidities other than hypertension benefits specifically, among all the patients who qualified for the infusion, there was an overall benefit of bamlanivimab infusion. The results of this study are encouraging and make the monoclonal antibody a good treatment option for COVID-19 patients with risk factors. The results of the BLAZE-2 trial support the use of bamlanivimab as well [10]. This study evaluated the efficacy of treating COVID-19 with bamlanivimab in nursing home patients and staff. The study concluded that bamlanivimab significantly reduced symptoms related to COVID-19 and could even lower the risk of contracting COVID-19. Although this study was conducted in a different healthcare environment in comparison to our study, they both show an association between treatment with bamlanivimab and improved patient outcomes. In future studies, other monoclonal antibodies or a combination of such treatment options can be explored to determine treatment benefit in this vulnerable patient population.

Study strengths

The two groups in our study were self-randomized even though our study was not a randomized control study. As data were collected after the fact, individual clinicians treating the patients were offering bamlanivimab equally to all qualified patients. Another strength was the equality in disease severity between the two groups. We did not include patients whose disease severity was so significant that they could not be discharged after the initial visit or required oxygen. By doing this, we were better able to see the impact bamlanivimab had on the ultimate disease course. Our study also used the population in a rural community hospital and as such our data apply to the everyday practicing emergency physicians who usually have to look at large university-based trials and apply them to their clinical setting.

Study limitations

Our study is a single-center study based in a rural community hospital with small sample size. The small sample size was the primary reason we could not better evaluate the possible mortality benefit of bamlanivimab. The follow-up in our study was by phone calls and chart review at 14 and 30 days by the pharmacy department as per their protocol. There could have been patients who were hospitalized in another facility in another city in either of the groups and could have not been accounted for in the hospitalized patients in either group.

## Conclusions

Although a much larger study is required to make more definitive recommendations, the findings of this study suggest that bamlanivimab infusion is a reasonable treatment option for patients with mild-to-moderate COVID-19 with at least one of the comorbidities required by the FDA indication. Given the current lack of treatment options for COVID-19 and the likelihood of COVID-19 being an ongoing presence for the foreseeable future, the possibility of bamlanivimab in reducing hospitalizations for the population of patients who are most at risk is very exciting and worthy of further exploration.
